# The Professional, Emotional, and Relational Experience of Psychologists Working With Adolescent Cancer Patients

**DOI:** 10.5334/cie.180

**Published:** 2026-02-13

**Authors:** Cristiana Punzi, Laura Guidotti, Paola Corsano

**Affiliations:** 1 Developmental Psychology, Department of Humanities, Social Sciences and Cultural Industries, University of Parma, Borgo Carissimi 10-43121 Parma, Italy; 2 Developmental Psychology, University Hospital, Via Gramsci-43121 Parma, Italy

**Keywords:** Pediatric psycho-oncology, psychologists’ emotional wellbeing, interdisciplinary care, care relationship

## Abstract

Despite the important role played by psychologists working with adolescent cancer patients in addressing adolescent-specific issues, there are few data concerning their professional, emotional, and relational experience. Using an open-ended questionnaire, this exploratory qualitative study examined the professional, emotional, and relational experiences of 20 psychologists (aged 27–66) working with adolescents in various Italian pediatric oncology units. Responses were analyzed through a step-by-step qualitative content analysis to identify recurring themes related to clinical practice, relational and intervention approaches, emotional experiences, and professional needs.

Findings revealed that participants provide psychological support through individualized, specific, and targeted interventions characterized by an empathic approach, active listening, and emotional closeness. Psychologists reported experiencing contrasting emotions (enthusiasm, compassion, and fulfillment, but also frustration, helplessness, and sadness), especially when dealing with issues such as loss of autonomy, disease progression, or death. Despite the emotional strain, most professionals perceived their work as meaningful and enriching. A need consistently emerged for continuous emotional processing, collegial dialogue, and supervision, to support the psychologists’ psychological wellbeing and strengthen multidisciplinary collaboration. Participants also emphasized the importance of targeted training to enhance their competence in working with adolescents with cancer.

Overall, the study highlights the lived experience and emotional landscape of psychologists working in pediatric oncology and underscores their contribution to adolescent care from a bio-psycho-social perspective. Supporting psychologists’ emotional resilience and professional growth is essential for promoting both clinician wellbeing and the quality of care provided to young patients.

## Introduction

Psychologists working with adolescent cancer patients in hospitals play a complex and multifaceted role as they attend to young patients during their hospitalization in the ward, in day-hospital care, and follow-up visits to monitor disease progression. As such, they are called upon to address a variety of challenges, such as identifying patient and family distress, promoting coping strategies, supporting adaptation to the disease, assessing its progression, and identifying dysfunctional and clinical psychological patterns. In addition, they integrate emotional aspects into clinical care and support the team, usually consisting of doctors and nurses, in recognizing their importance, thus promoting a deeper understanding of the psychological experiences of patients and their families.

These demands are made especially challenging by the developmental stage of adolescent patients. Adolescence involves deep physical, psychological, and social changes, and the onset of cancer at this stage disrupts developmental tasks across emotional, cognitive, relational, and educational domains (Barr et al., 2016; Boufkhed et al., 2023; Thomas et al., 2010). In this sense, adolescence represents a unique and particularly sensitive developmental phase, in which illness not only affects physical health but also interferes with processes of identity formation, autonomy, and social belonging, making the psychologist’s role both complex and crucial.

The psychological and social needs typical of adolescence, amplified and made more salient by the onset of illness, can be supported by the work of a psychologist. Based on the bio-psycho-social model, which considers biological, psychological, and social factors, psychological support is an integral component of multidisciplinary care. Within this framework, the bio-psycho-social model serves as the conceptual foundation of this study, emphasizing the interdependence between the emotional, cognitive, and relational dimensions of care and the systemic collaboration among professionals, whereby different professionals collaborate to promote the psychological and behavioral wellbeing of young patients and to prevent dysfunctional outcomes in the short, medium, and long term (Pagani Bagliacca et al., 2020; Ricadat et al., 2019). Particularly interesting is the work that psychologists can carry out jointly with hospital teachers, who often deal directly with the emotions expressed by patients, carry out psychoeducational activities aimed at improving their wellbeing, and reduce negative emotions, distress, and pain (Ciucci et al., 2024). Working directly with patients’ emotions can be particularly challenging for both types of professionals, who, therefore, often share similar emotional experiences and needs (Caggiano et al., 2021; Magalhães et al., 2018; McNamara, 2024).

Although research on stress and burnout resulting from work in oncology is extensive, it mainly concerns physicians and nurses (Font et al., 2015; Girgis et al., 2009; Kushal et al., 2018), and even when psychologists are considered, their experience is investigated alongside that of other health professionals, not as a separate issue (Eelen et al., 2014; Girgis et al., 2009). As such, more than 30 years after the birth of psycho-oncology and its establishment in the hospital setting (Breitbart et al., 2021), there are still few data on the specific emotional and relational experience of psychologists working with adolescent cancer patients (Patenaude et al., 2011; Wiener et al., 2012).

Among the existing studies, the research conducted by Wiener and colleagues (2012) was the first international, multidisciplinary investigation into the work experiences of psychologists in pediatric psycho-oncology. Among their findings, the authors highlighted a susceptibility to stress in the care setting caused by limited resources, heavy workload, and poor team cohesion. In particular, psychologists in the pediatric oncology department perceived the following areas as especially challenging: interprofessional relationships, lack of supervision, and the need for additional specialized training (Patenaude et al., 2011; Wiener et al., 2012). In addition, anger, guilt, frustration, and emotional exhaustion are often experienced by psychologists who provide therapeutic support to adolescents with unfavorable prognoses (limited life expectancy) in a psycho-emotional spiral characterized by feelings of helplessness, depersonalization, and low personal accomplishment and self-efficacy, which can lead to detachment or even professional withdrawal (Eelen et al., 2014; Girgis et al., 2009).

However, Wiener et al. (2012) also found reports of rewarding elements of working in an oncology department, including positive relationships with some colleagues and personal growth in the care setting, highlighting the ambivalence of working in pediatric oncology, where distress and fulfilment coexist within a demanding emotional landscape. Moreover, the authors emphasized that psychologists could benefit from specific training courses on clinical and communication interventions and supervision.

To the best of our knowledge, in the wake of Wiener and colleagues’ research, no international studies have been conducted that focus specifically on the professional experiences of psychologists working with adolescent cancer patients. In Italy, only one study (Cavalli & Bovero, 2015) has investigated the psychologist’s emotional resonance in relation to oncological minor patients in a pediatric department. In their analysis of a clinical case involving a 14-year-old girl with cancer, the authors underscored the significance of establishing a therapeutic alliance grounded in empathy and shared suffering. This approach fosters trust and encouragement, facilitating the expression of emotions by the patient and the psychologist, which can then be addressed to promote greater wellbeing.

Although there is a clear lack of research on the experiences of psychologists working with cancer patients, guidelines exist in support for these professionals, aimed at increasing their skills when assisting patients to achieve optimal psychological health during and after treatment for cancer and to manage their own emotional wellbeing when working with such a population (Zomerdijk et al., 2023). Nevertheless, the limited data available underline the need to explore in-depth how psychologists experience their work with adolescents with cancer, both at the emotional and relational level, and how they can be better supported within multidisciplinary care systems (Turnell et al., 2016).

## The Present Study

This exploratory study sought to deepen understanding of psychologists’ experiences in pediatric oncology. Specifically, it investigated the professional, emotional, and relational dimensions of psychologists working with adolescent cancer patients, considering the complexity that characterizes this developmental stage and the hospital context. Grounded in Engel’s (1977) bio-psycho-social model, which conceptualizes health and illness as outcomes of the dynamic interaction among biological, psychological, and social systems, the study adopted a systemic and integrative perspective that frames psychological work as an essential component of holistic care. Within this framework, psychologists are viewed as key actors in the network of care, whose experiences emerge from the continuous interplay between personal emotions, professional competencies, and team-based relational dynamics. This approach enables a deeper understanding of how psychologists contribute to patients’ overall adjustment and wellbeing, while simultaneously navigating the emotional and organizational demands of the hospital environment.

To capture the emotional and relational nuances of psychologists’ experiences, which cannot be easily quantified, we chose a methodological approach that emphasized qualitative analysis, with the objective of addressing three primary research questions:

(RQ1): How do ward psychologists work with adolescent cancer patients? What psychological support do they provide and how does their patients’ developmental stage influence their work?

(RQ2): What emotional and relational experiences do psychologists have when working with this patient population?

(RQ3): What support do they need in this kind of work?

In short, the study sought to answer the overarching question: How do psychologists perceive, experience, and manage the emotional and relational challenges of working with adolescent cancer patients within the hospital care context?

## Methods

### Participants

All pediatric oncology hospitals departments in Italy (*N* = 50) were identified through the following website: https://www.aieop.org/web/chi-siamo/. The facilities were then contacted directly to get in touch with psychologists by presenting and explaining the aims of the study. As a result, 50 psychologists were contacted, of whom 20 (5 males and 15 females), aged 27–66 years (*M* = 45.37; *SD* = 9.29) accepted to participate. They received a research prospectus and a link to directly access an open-ended questionnaire available on an online platform (Microsoft Forms). All the participants gave their informed consent, and the study was conducted according to the Declaration of Helsinki, the guidelines on “Good Practice in Research and in the Publication and Dissemination of Results” issued by the authors’ university, and the Code of Research Ethics by the Italian Psychology Association. Participants were guaranteed anonymity, and all the data were collected and coded to protect privacy.

The geographical distribution of the participants is as follows: 64% currently work in hospitals in northern Italy, 25% in southern Italy, and 11% in central Italy. Most participants (*n* = 15) hold a specialization diploma in psychotherapy, while 5 have a master’s degree in psychology. Their years of experience in the oncology field range from 5 to 30 years (*M* = 13.5; *SD* = 8.58).

### Instruments

Based on the professional experience of the researchers and the existing literature (Patenaude et al., 2011; Wiener et al., 2012), and considering the research questions, an open-ended questionnaire was constructed (see Supplementary Material 1), aimed at investigating the psychologists’ emotional and relational experience of working with an adolescent cancer patient in a pediatric oncology department. The questionnaire was not pretested but was reviewed several times by experts before being offered to participants.

The tool consisted of 27 items organized in several thematic areas: the psychologist’s (a) work with the oncological adolescent, (b) emotional experience, and (c) needs. Five questions gathered socio-demographic information, including age, gender, educational background, geographical location of the hospital, and years of experience in the pediatric psycho-oncology field. Three questions explored the general characteristics of the participants’ professional activity. Ten questions focused on participants’ emotional experiences while working with adolescent patients, and two items examined their behavioral reactions and interactions with these young patients. Three questions addressed the difficulties encountered in their professional practice, and the final four items aimed to evaluate therapeutic work as a whole. All the participants answered every question in the questionnaire.

### Procedures and Data Analysis

A qualitative content analysis was conducted to identify and describe psychologists’ experiences in working with adolescent cancer patients and explore their perceived needs. The analytical procedure was conceptually inspired by Burnard’s (1991) step-by-step model, but it was not applied in its entirety. A deductive analytical approach was adopted, allowing the researchers to organize the data according to predefined thematic areas derived from the research questions. The team consisted of a developmental psychology professor, a psychologist, and a clinical psychologist with many years of experience in the field of pediatric oncology. The authors’ background knowledge stemmed from their academic and professional experiences in hospital settings.

The researchers read through the material multiple times to become immersed in the data, and the subsequent analysis was carried out gradually and progressively. The entire research team also participated in the analysis and discussion of the results. The analytic process followed four interrelated phases: familiarization, coding, theme development, and interpretation. All the researchers (the authors of this article) read the responses repeatedly to achieve a deep familiarity with the data and to capture their emotional tone and meaning. Initial codes were then generated independently by each author, focusing on patterns of meaning relevant to the study aims. The research team subsequently compared and discussed the emerging codes to refine categories and ensure coherence. Discrepancies were resolved through discussion and collective reflection, enhancing the credibility and consistency of the findings.

Through an iterative process of comparison and synthesis, broader themes were developed that reflected the emotional, relational, and professional dimensions of the psychologists’ experiences. Codes and themes were archived/recorded and organized using Microsoft Excel, which facilitated systematic review and cross-case comparison. The final interpretation was reached through team consensus, integrating both data-driven insights and the researchers’ professional expertise in pediatric oncology settings.

## Results

### Nature of Work With Adolescent Cancer Patients

The first research question addressed how ward psychologists work with adolescent cancer patients, what psychological support they provide, and how the developmental stage of adolescence influences their work.

#### Psychological Support Provided

All the participants described their primary task as offering psychological support to adolescents with cancer and their families throughout all the disease stages, from the communication of diagnosis to treatment and follow-up. Their work focused on helping patients adapt to the illness and its consequences in emotional, relational, and cognitive terms.

Different methodologies were employed, for example, the active listening technique, which requires close attention to what the patient is saying in order to gain an accurate understanding of the narrative. Other interventions were based on play-recreational activities, traceable to art therapy, photography workshops, reading and storytelling (*n* = 7), group meetings (*n* = 7), and use of testing tools (*n* = 3). Also, relaxation techniques, biofeedback, and EMDR (Eye Movement Desensitization and Reprocessing) were used (*n* = 4). Moreover, four respondents explicitly stated that their work was also aimed at the whole family.

#### Working With and Approaching Adolescents

The experience of working with adolescents was described as both rewarding and challenging. On one hand, eight psychologists reported fewer difficulties in their therapeutic interactions, attributing this to adolescents’ increased maturity (*n* = 3), greater capacity to seek help (*n* = 2), and reduced parental interference (*n* = 3). These factors are believed to support the development of a positive therapeutic alliance and facilitate more direct and meaningful communication with the young patient. On the other hand, nine professionals emphasized the challenges of engaging with adolescents, citing the complexity of their developmental phase. As one participant explained:

“Formal operational thinking makes the cognitive asset more complex, and the disease creeps into the process of personal identity construction, hindering adolescents’ efforts to achieve autonomy, emancipation and conduct future planning.” (n. 8)

Moreover, the presence of more structured defense mechanisms in adolescence can heighten resistance toward the psychologist, making therapeutic engagement more difficult. Some professionals emphasized the importance of involving the family to foster safety and strengthen the therapeutic bond with the adolescent. One noted the unique challenge of working with individuals who do not fit neatly into the child or adult categories, which requires constant flexibility in the therapeutic approach. While only three participants attributed the level of difficulty primarily to individual therapist characteristics, underscoring the absence of a universal approach, one participant identified a specific difficulty in the therapeutic relationship; namely, a struggle that is more psychological than physical. Reflecting on their clinical practice, 17 participants reported frequent engagement with thoughts about their work outside the therapeutic setting, while others considered this reflection to be of marginal importance.

In parallel, 11 professionals highlighted the challenge of establishing initial contact with adolescents, difficulties they attributed not only to the developmental stage of adolescence but also to personal characteristics and the evolving nature of the illness. Despite these difficulties, the psychologists described adopting an approach characterized by authenticity, spontaneity, and emotional openness. They sought to build relationships grounded in reciprocity and acceptance through genuine curiosity, active listening, and the use of language that resonates with the adolescent’s experiences and developmental perspective. As one participant said:

“I put myself across in a simple and natural way, first trying to get to know him better regardless of the experience he was going through. I approach him slowly, answer his questions, and gradually get to know him also about aspects related to the disease, treatments, friends, relationship with parents, relationship with doctors … I try to observe him with “normal” eyes without being judgmental, but also embracing his frailty.” (n. 18)

Furthermore, in response to the negative emotions expressed by the adolescent, most professionals recognize the importance of listening, acceptance, and containment, both physical and psychological, in the relationship with the adolescent. In this regard, it was found that almost all participants reported using an approach based on closeness and understanding with the patient, with the goal of reprocessing the emotions they are experiencing.

### Emotional Experience

The second research question focused on the emotional and relational experiences that psychologists feel when working with cancer adolescent patients and their families.

#### Emotions in Relationships With Patients

Five psychologists reported experiencing positive emotions, including enthusiasm, curiosity, interest, tenderness, compassion, and complicity, in their professional interactions with adolescent patients. These feelings indicate a pervasive sense of fulfilment derived from working with this population. Only one of the respondents indicated that they experienced only negative emotions. including anger, sadness, fear, frustration, and helplessness. Regarding frustration in the relationship with adolescent cancer patients, 15 reported experiencing this feeling in various circumstances, such as when patients are opposed to or reject professional and psychological support, when an adequate means of communication is not found, and when there are long periods of silence from patients. Furthermore, some respondents noted experiencing this feeling when a relapse or progression of the disease occurs. Three psychologists stated that they did not experience this feeling with this patient group, and 2 stated that it occurred very rarely. Moreover, many psychologists (11) reported experiencing both positive and negative emotions; while 3 participants provided responses classified as neutral, as no emotional connotation was present.

“I feel similar emotions to those of working with adults.” (n. 5)

In general, the relationship with adolescents was found to be both stimulating and challenging, providing psychologists with a daily opportunity to engage with new stimuli.

“Overall, positive emotions clearly predominate, essentially related to the sense of wonder of getting to know growing human beings dealing with important life issues.” (n. 17)

#### Emotions in Reference to Specific Themes

Specific areas typical of adolescence were also explored: bodily changes, sexuality, romantic relationships, social transitions, the future, and death. Participants provided a series of responses that revealed their emotional experiences regarding these topics.

**Body Changes**. The professionals reported experiencing a range of emotions and feelings, including embarrassment, tenderness, empathy, sorrow, anxiety, fear, and sadness, and understanding for the distress manifested by the young patient related to body changes.

“I know that these consequences are more important to them than other ones, perhaps objectively more serious, such as the long-term effects of chemotherapy. But I understand that for them hair and image in general represent important points in the relationship with the self and the other.” (n. 3)

**Sexuality**. Six professionals indicated that they seldom or never discussed sexual matters with the young patients, as such conversations often evoke negative emotions such as discomfort, anxiety, embarrassment, or concern about the subject being inappropriate. Furthermore, two respondents were able to address this issue calmly, claiming that they did so by establishing a bond with the patient. Only one of the psychologists reported not experiencing specific emotions in relation to this topic.

**Sentimental Relationships**. Talk about relationships usually evokes positive emotions, such as happiness, involvement, serenity, joy, and complicity, as they represent a sense of normality within the turmoil of illness. Talking about them also reflects the adolescent’s trust in the psychologist. However, five psychologists reported experiencing negative emotions when the relationship became a source of distress for the patient.

“In this case there are heightened experiences of injustice, loss. But sometimes also awe and wonder at the strength and resources that can emerge from teenage romantic relationships.” (n. 17)

**Social Change**. The impact of illness on social relationships, at a stage of development when these become crucial, was a topic that evoked a range of emotions among the participants. While the majority (*n* = 11) reported experiencing negative emotions such as sadness, sorrow, discomfort, worry, and anger, it is noteworthy that in some instances, these negative emotions were accompanied by positive feelings and thoughts (*n* = 6).

“It’s a subject I touch on so much with them, sometimes thorny and not easy. However, I feel at ease when we talk about it together, even if I am a little careful not to fall into clichés or give ready-made answers.” (n. 17)

**The Future**. This topic was addressed by all participants, who generally reported that they preferred to talk about concrete projects (e.g., journeys, studies) in both the short and long term, rather than about the future in an abstract sense. They claimed they encouraged adolescent patients to mobilize psychological and emotional resources, fostering activation and hope for the future. However, sometimes professionals are reluctant to offer false hope regarding the course of illness, or to encourage feelings of frustration and anger as the prospect of the future is contrasted with the fatigue experienced in the present.

“It often happens that we talk about the future; the uncertain future is the driving force of therapeutic work. We talk about future journeys, desires to be fulfilled, new relational perspectives gained during treatment, new awareness that provides for different life horizons than before. It is a type of narrative that arouses a lot of enthusiasm in me, but also the fear of creating inappropriate expectations for the disease pathway.” (n. 2)

**Death**. This is a subject that most psychologists (*n* = 16) approach, albeit with difficulty, but with the understanding that it should not be a taboo. The professionals reported experiencing a range of negative emotions when dealing with this topic, including anxiety, fear, sadness, a sense of helplessness and anger. They preferred to talk about death in general, without referring specifically to their patients’ condition, and to talk about it when they recognized that adolescents needed to talk about it; for example, after the death of other patients or fear at facing death themselves. In some cases, however, when faced with a patient’s worsening illness, respondents reported feeling forced to address the issue directly.

“It can happen to talk about death with respect to what emerges from the news they have about the progression of the disease. The arguments focus on the life projects that the person intends to carry out and that they fear they will not be able to complete. It arouses emotions of compassion, sadness, sense of injustice, helplessness.” (n. 8)

#### Emotions Related to the Death of the Adolescent Patient

Psychologists sometimes have to deal with death not only as a subject of dialogue with patients, but as event that actually occurs. The death of a patient creates a significant gap in the professional’s life, inevitably characterized by negative emotions, including grief, sadness and anger. The predominant emotional state is sadness accompanied by anger. Additionally, a sense of helplessness and defeat is frequently observed. Nevertheless, over time, most professionals working in this field adopt a constructive approach to these experiences, leading to a sense of motivation to continue in their profession and a feeling of providing further support to parents of the deceased patient. One psychologist emphasized the value of sharing their experiences with their colleagues or during supervision sessions.

“Yes. Many times, and each time I felt anger, pain, impotence. Accompanying a young boy/girl towards the end of life is a very emotionally demanding experience for the therapist as well. It is important to share these aspects with colleagues or in supervision.” (n. 15)

Another psychologist reported a sense of detachment from reality for a period of several days following the death of a patient, while continuing to perform their usual duties. However, in some cases, the psychologists experienced a sense of relief following the death of a patient who had been suffering for a lengthy period. In only one case had the experience of a young patient’s death not affected the practitioner’s career.

#### Emotions in the Relationship With Caregivers

Work with adolescent patients necessitates the involvement of parents, which can also elicit a range of emotional responses, both positive and negative, in psychologists. Positive emotions include feelings of empathy, satisfaction, serenity, admiration, and tenderness. Negative emotions, on the other hand, are predominantly manifested as sadness, anger, and fear, coupled with frustration, anxiety, a sense of injustice and helplessness. Three psychologists emphasized that the range of emotions regarding their relationship with caregiver is very wide and depends on several factors, making it difficult to identify and point to a single one. Those who reported difficulties and negative emotions when working with adolescents reported the same feelings in their relationships with the parents. This is due to the defensiveness and resistance they may show towards the psychologist or their attempts to interfere in the therapeutic work, which makes it more difficult to establish a good therapeutic alliance.

“Parents may perceive psychological support during hospitalization as an unwelcome intrusion. I find that I must adapt the interventions according to the defenses not only of the adolescent but also of the parents. The therapeutic alliance with the family system is harder to establish.” (n. 9)

### Psychologists’ Needs

Finally, the last research question addressed the psychologists’ needs. Most professionals consider managing their own emotions essential to maintaining psycho-emotional wellbeing and working effectively within the multidisciplinary healthcare team. Eleven of the psychologists in the current study identified a need to discuss matters with their colleagues and the multidisciplinary staff in the hospital.

“The possibility to discuss cases with other psychologists or other members of the medical-nursing team, perhaps even with an educator figure who can also conduct psycho-educational plays and not only psychological interviews.” (n. 18)

They also highlighted the value of increasing opportunities for supervision, support, and debriefing. Furthermore, three professionals emphasized the importance of specific training aimed at deepening their understanding of the issues related to their patients. Only three participants reported no specific needs. Overall, over findings offer a comprehensive, concise overview of psychologists’ professional, emotional, and relational experiences in pediatric oncology, addressing the study’s three research questions.

Regarding RQ1, psychologists’ interventions emerged as highly individualized and developmentally attuned, combining clinical and creative strategies to foster adolescents’ adjustment and emotional wellbeing.

For RQ2, psychologists experienced a blend of positive and negative emotions (empathy and fulfillment, alongside sadness and helplessness) which, albeit demanding, also serve as sources of personal and professional growth.

Concerning RQ3, participants emphasized the need for supervision, emotional reflection, and peer collaboration, together with ongoing training to sustain wellbeing and professional effectiveness.

Taken together, these themes portray the psychologist’s role in adolescent oncology as a balance of technical competence, emotional involvement, and reflective practice, aimed at promoting the wellbeing of both patients and professionals within a bio-psycho-social framework.

## Discussion

This study provides new insights into the experience of psychologists working with adolescents with cancer, an important, yet understudied field. The aim was to explore their emotional and relational experiences, as well as their professional challenges and needs. The qualitative analysis of open-ended questionnaire responses made it possible to identify psychologists’ working methods, their emotional engagement with patients and families, and their perceived needs for support and training.

With regard to the psychologists’ working methods, all the participants shared the common goal of providing psychological support to help adolescents adjust to illness, although their approaches reflected different professional backgrounds. The clinical interview emerged as the most widely used tool, fostering therapeutic alliance and open communication. Alongside traditional methods, several psychologists reported employing creative techniques such as art, group, or play-based activities to align with adolescents’ interests and developmental characteristics, highlighting the importance of tailoring interventions to suit the heterogeneous needs of young patients (Li et al., 2024; Thornton et al., 2020). From an interpretative perspective, this finding suggests that psychologists working in pediatric oncology need to integrate developmental sensitivity with clinical expertise, adopting a flexible and reflective attitude that allows them to modulate their approach according to each adolescent’s stage of autonomy and identity formation.

Moreover, working with adolescents was described as both rewarding and demanding. About half of the participants viewed it as less difficult than working with younger patients, citing adolescents’ maturity and autonomy as facilitating factors; others emphasized greater challenges related to resistance, identity development, and family dynamics. As Cavalli and Bovero (2015) noted, each therapeutic relationship is unique and shaped by the characteristics of all the parties involved.

Building on these professional approaches, the study revealed the intense emotional dimension that characterizes psychologists’ daily work with adolescents. The emotional experience appeared complex and multifaceted: bodily changes and emerging sexuality often elicited discomfort or sadness, whereas discussions of romantic relationships were associated with positive emotions such as joy and complicity. This contrast likely reflects both the sensitivity of sexual issues during adolescence and adolescents’ difficulties in talking about them.

Overall, the psychologists reported intense emotional participation in their patients’ experiences, displaying empathy in moments of suffering and joy alike, and their ability to regulate emotions and perceive themselves as effective emerged as a key factor in shaping their professional wellbeing. Particularly challenging were conversations with patients and their families about the future and death. All the participants reported addressing the future during sessions, consistent with Moore et al. (1969), who emphasized the importance of nurturing hope while acknowledging uncertainty. Death, though recognized by participating psychologists as an unavoidable topic, was typically discussed only when adolescents initiated the conversation, as it aroused powerful emotions such as sadness, fear, and inadequacy. This reluctance may reflect, as Moore et al. (1969) suggested, the tendency among healthcare professionals to perceive patient death as a personal failure.

Furthermore, psychologists’ emotional involvement often extended beyond the workplace, with many continuing to think about their patients outside working hours, a phenomenon that previous studies have shown to be both a sign of dedication and a source of emotional strain (Eelen et al., 2014; Kovács et al., 2010). Excessive workload, emotional demands, and lack of resources such as social support or supervision can contribute to burnout and a reduced quality of care. In this regard, these findings highlight the need to integrate end-of-life communication and emotional coping within continuing education and supervision programs. Structured spaces for reflective dialogue should be promoted as institutional resources that enable psychologists to process grief, prevent emotional exhaustion, and transform distressing experiences into learning opportunities that enhance therapeutic presence. Hospitals and clinical centers could thus enhance psychologists’ wellbeing and professional growth by implementing regular debriefing sessions, peer supervision, and interdisciplinary discussion groups, fostering both preventive and developmental functions within psycho-oncological practice.

Despite these intense emotional challenges, participants demonstrated strong coping strategies and generally derived meaningful fulfilment from their work. They appeared to possess significant internal and external resources, often finding gratification and enthusiasm in their relationships with adolescents even in difficult circumstances. This finding, consistent with Turnell et al. (2016) and Wiener et al. (2012), suggests that positive and negative emotions can coexist as part of a growth-oriented professional experience and that the availability of resources, such as supervision and collegial discussion, is crucial for moderating stress and sustaining motivation.

Responses to the final item of the questionnaire revealed professionals’ main needs: Opportunities for emotional reflection, peer exchange, and interdisciplinary teamwork were among the most frequently cited, while several participants also emphasized the importance of ongoing training to remain updated on adolescent development and psycho-oncological issues. These findings align with Wiener et al. (2012), who noted that limited staff and high workload in pediatric psycho-oncology often restrict opportunities for peer support and supervision, increasing professional isolation. Building collaborative networks among mental health professionals could thus enhance emotional management, methodological exchange, and the overall quality of care.

Taken together, these findings underscore the broader implications of psychologists’ experiences for pediatric oncology practice. Within this context, psychologists play a crucial role in integrating emotional and relational care into the multidisciplinary setting. As previous studies confirm (Caggiano et al., 2021; Eelen et al., 2014; Girgis et al., 2009), healthcare professionals working with young cancer patients often experience emotional distress; however, psychologists can transform this experience into an opportunity for personal and professional growth through supervision, training, and collaboration. They can also contribute to psychoeducational programs in partnership with teachers and educators (Magalhães et al., 2018), promoting more comprehensive support for hospitalized adolescents (Małkowska-Szkutnik et al., 2021; McNamara, 2024). From a bio-psycho-social perspective, strengthening integrated and multidisciplinary care systems represents an essential step toward improving the perceived quality of care and satisfaction among patients and families (Baxter et al., 2018; Marshall et al., 2022).

## Limitations and Future Directions

Despite the importance of its findings, this exploratory study has some limitations. The first is related to the difficulty of contacting psychologists working in pediatric oncology departments, which made it possible to recruit only a small sample and, therefore, prevented us from generalizing the results.

Further criticism can be made of the online completion of the questionnaire, which did not allow for a direct exchange with participants. Furthermore, potential self-selection bias may have occurred; that is, psychologists who agreed to participate might differ systematically from those who did not. For example, those experiencing extreme burnout might have declined. In addition, the use of an open-ended questionnaire may be subject to the social desirability of the participants. Finally, in reference to the instrument, it is important to mention that there is a significant imbalance in the different sections. For example, there are 10 questions on emotional experiences but only 3 on behavioral reactions and interactions.

## Conclusions

Despite its limitations, this qualitative study sheds light on the emotional complexity of psychologists working with adolescent cancer patients and their need for greater peer discussion, collaboration, and institutional support to enhance both professional effectiveness and emotional wellbeing. By focusing specifically on psychologists, a group of professionals often overlooked compared to physicians and nurses (Escot et al., 2001; Kuerer et al., 2007), the study underscores the unique nature of their emotional involvement and the added value of their role within multidisciplinary care teams.

From a practical standpoint, the findings point to the importance of implementing structured opportunities for reflection and supervision within hospital settings. For example, regular discussion groups or focus groups, possibly facilitated by a moderator, could serve as safe spaces where professionals share experiences, elaborate emotional challenges, and collectively develop strategies to prevent burnout. Such initiatives may contribute not only to sustaining psychologists’ mental health and job satisfaction but also to improving the overall quality of care and relationships with patients and families.

From a research standpoint, future studies could further explore concrete ways to support psychologists in oncology contexts, including examining subjective perceptions of workload and identifying effective organizational and training interventions. Extending this line of inquiry with larger and more diverse samples would enhance generalizability and foster the development of evidence-based strategies to strengthen emotional resilience, professional competence, and interdisciplinary collaboration in pediatric psycho-oncology.

## Additional File

The additional file for this article can be found as follows:

10.5334/cie.180.s1 Supplementary File 1. Questionnaire Administered to Psychologists Working in hospital Settings With Adolescents With Cancer.
