# Supplementary material for: The Professional, Emotional, and Relational Experience of Psychologists Working With Adolescent Cancer Patients
Source: Contin Educ. 2026 Feb 13;7(1):12–24. doi: 10.5334/cie.180 (PMC12904134; doi:10.5334/cie.180)
Supplement: Supplementary File 1. — Questionnaire Administered to Psychologists Working in hospital Settings With Adolescents With Cancer. [file cie-7-1-180-s1.pdf]

Psychologists Working With Adolescent Cancer Patients: Exploring Their Emotional and Relational Experience*

Supplementary Material 1

Cristiana Punzi, Laura Guidotti and Paola Corsano

The Questionnaire

**GENERAL INFORMATION**
Age
Gender
Educational level
Geographic area where you are working
How many years have you worked in pediatric oncology?
**THE PSYCHOLOGIST'S WORK WITH THE ONCOLOGICAL ADOLESCENT**
What does your work with the adolescent consist of?
Is working with adolescents more strenuous than working with younger children? Justify your answer.
What tools do you use in your therapeutic relationship with the adolescent?
**EMOTIONAL EXPERIENCE**
In general, what emotions does working with adolescents arouse in you?
What emotions do you feel in relating to the adolescent’s parents?
How do you react emotionally to emotions expressed by the teenager, such as anger, anxiety, sadness, fear, shame?
What emotions do you feel when dealing with your teenager on topics regarding bodily changes related to illness?
What emotions do you feel in dealing with your adolescent on topics regarding sexuality during the period of illness?
What emotions do you feel in dealing with the teenager on topics concerning romantic relationships during the period of illness?
What emotions do you feel in dealing with your adolescent on topics concerning social changes deriving from the experience of illness?

The original article can be downloaded from https://doi.org/10.5334/cie.180

Do you ever talk about the future with your teenager? If so, with respect to what? What emotions does it arouse in you?
Do you ever talk about death with your teenager? If so, in relation to what? What emotions does it arouse in you?
Have you ever had to deal with the death of an adolescent patient with whom you had worked? If so, what did you feel?
**BEHAVIORAL REACTIONS**
How is your relationship with the adolescent?
How do you react behaviorally to emotions expressed by the adolescent, such as anger, anxiety, sadness, fear, shame?
**DIFFICULTIES**
What difficulties do you perceive in your relationship with the adolescent?
Do you sometimes experience frustration when working with the adolescent? Justify your answer.
Outside of department work, on a scale of 0-10, how much do you think about the working and emotional relationship you have with a particular teenager?
**OVERALL ASSESSMENT OF THE THERAPEUTIC WORK**
In terms of your professional experience, describe a positive aspect of working with adolescents.
In terms of your professional experience, describe a negative aspect of working with adolescents.
On a scale of 0-10 how much satisfaction does working with adolescents give you?
Do you feel that you need something in order to better the experience of the therapeutic relationship?

*Translated from Italian.
